# Working Memory Phenotypes in Early Multiple Sclerosis: Appraisal of Phenotype Frequency, Progression and Test Sensitivity

**DOI:** 10.3390/jcm11102936

**Published:** 2022-05-23

**Authors:** Meaghan Clough, Jade Bartholomew, Owen B. White, Joanne Fielding

**Affiliations:** Department of Neurosciences, Central Clinical School, Monash University, Alfred Centre, 99 Commercial Rd., Melbourne, VIC 3004, Australia; jade.bartholomew@monash.edu (J.B.); owen.white@monash.edu (O.B.W.); joanne.fielding@monash.edu (J.F.)

**Keywords:** multiple sclerosis, working memory, working memory assessment, cognition, ocular motor, early multiple sclerosis

## Abstract

Working memory (WM) impairments are common and debilitating symptoms of multiple sclerosis (MS), often emerging early in the disease. Predominantly, WM impairments are considered in a binary manner, with patients considered either impaired or not based on a single test. However, WM is comprised of different activated subcomponents depending upon the type of information (auditory, visual) and integration requirements. As such, unique WM impairment phenotypes occur. We aimed to determine the most frequent WM phenotypes in early MS, how they progress and which WM test(s) provide the best measure of WM impairment. A total of 88 participants (63 early relapsing–remitting MS: RRMS, 25 healthy controls) completed five WM tests (visual–spatial, auditory, episodic, executive) as well as the symbol digit modalities test as a measure of processing speed. RRMS patients were followed-up for two years. Factors affecting WM (age/gender/intelligence/mood) and MS factors (disease duration/disability) were also evaluated. Some 61.9% of RRMS patients were impaired on at least one WM subcomponent. The most subcomponents impaired were visual,–spatial and auditory WM. The most common WM phenotypes were; (1) visual–spatial sketchpad + episodic buffer + phonological loop + central executive, (2) visual–spatial sketchpad + central executive. The test of visual–spatial WM provided the best diagnostic accuracy for detecting WM impairment and progression. The SDMT did not achieve diagnostic accuracy greater than chance. Although this may be unsurprising, given that the SDMT is a measure of cognitive processing speed in MS, this does highlight the limitation of the SDMT as a general screening tool for cognitive impairment in early MS.

## 1. Introduction

Multiple sclerosis (MS) is a chronic disease of the central nervous system, characterised by an interplay between neurodegenerative and inflammatory processes. Cognitive impairment is reported in up to 70% of people with MS (pwMS) irrespective of disease subtype [1]. Changes to working memory (WM) are common, affecting the proficiency with which pwMS maintain and manipulate different types of information in an “active” and accessible format [2,3,4]. This in turn impacts higher cognitive processes associated with goal-directed behaviour [5]. However, despite this general understanding of WM impairment in MS, there is little understanding how WM impairments differentially manifest within individual pwMS (WM phenotypes), particularly early within the disease. Indeed, WM is thought to rely upon discrete subcomponents activated depending upon the type of information (auditory, visual) and requirement for maintenance and integration. Given the known pathophysiological changes that are frequently evident on Visual Evoked Potential (VEP) and Auditory Evoked Potential (AEP) investigations, it is likely that discrete changes to WM processing occur [6,7]. This knowledge gap has ramifications for developing efficacious treatments or interventions/rehabilitation strategies for WM, with success thought to be maximised when the specific phenotype is targeted, appropriate tests are selected [8,9], and intervention occurs early when neural compensatory mechanisms are still active [10].

A prominent WM theory by Baddeley and Hitch [11,12] posits that WM consists of four specialised subcomponents that form a widely dispersed, functionally integrated, yet partially segregated (according to subcomponent) neural network [13]. The phonological loop and visuospatial sketchpad subcomponents, actively (rehearse) and passively maintain auditory and visual–spatial information, respectively. The episodic buffer subcomponent represents a storage system that integrates newly acquired information from the visual–spatial sketchpad and/or phonological loop with long-term memory. Lastly, a central executive subcomponent coordinates the aforementioned subcomponents, controlling the focus of attention to maximise the proficiency of information maintenance and integration with other cognitive processes [11,12,14]. The capacity of each subcomponent is considered variable, both within and between individuals, a consequence of modifying factors such as genetics, age, gender, intelligence, mood, and, in the case of disease, disease-specific factors such as disease duration and severity [15,16,17]. Given the widespread nature of MS pathology and the heterogeneity observed between pwMS, it is likely that there are individual differences in the WM subcomponents impaired.

WM impairments have been reported in pwMS across all subcomponents, although they are most frequently associated with the episodic buffer [1,2,18,19,20]. However, in early MS, impairments appear to primarily involve the visual–spatial sketchpad and phonological loop, with some reports suggesting a sparing of the executive and episodic buffer [21,22]. This also appears to be the case for those with a clinically isolated syndrome [17,22,23,24], which is the first clinical manifestation of MS in approximately 85% of patients [21]. To our knowledge, only one study has examined different WM subcomponents within a single study in pwMS; however, it only examined patients with significant disability (Expanded Disability Status Scale: EDSS ~4) and advanced disease duration (~10 years) [19]. Results of this study are largely in line with previous studies, with the episodic buffer most severely impaired. What it is unclear, however, is the frequency with which subcomponent impairments co-occurred within patients, what effect disease severity and duration had, and whether different subcomponent impairments differentially progressed once disease-modifying and individual factors are considered.

The aim of our study was to investigate different WM subcomponent in pwMS with early relapsing remitting (RRMS) disease (EDSS < 2) and determine: (1) the WM phenotype(s) most frequently affected in early RRMS and whether phenotypes most commonly implicate single or multiple subcomponents, (2) how different WM subcomponents progress over time, accounting for individual and disease-modifying factors, and (3) which test(s) provide the best diagnostic measure of WM impairment and measures progression. It was anticipated that pwMS would exhibit a greater frequency of impairment associated with the visual–spatial sketchpad and phonological loop. Furthermore, it was anticipated that impairment associated with each WM subcomponent would progress at a different rate over the study period, with certain tests offering different levels of sensitivity to progression and WM diagnosis.

## 2. Materials and Methods

### 2.1. Participants

A total of 63 patients, 14 with a clinically isolated syndrome (CIS) and 49 with clinically definite MS (CDMS) diagnosed as early relapsing–remitting MS (RRMS, EDSS < 2) were included. All CDMS patients were diagnosed based on the McDonald criteria 2017 revision, and all CIS patients had an initial neurological event with MRI abnormality consistent with demyelination. No patient had experienced a clinical event within 60 days prior to testing. All CDMS patients were taking disease-modifying treatments throughout the course of the study (90% Fingolimod, 5% Cladribine, 5% Alemtuzumab). Some 60% of CIS patients were taking disease-modifying treatments throughout the course of the study (95% Fingolomod, 5% Cladribine). No patient was treated with corticosteroids within 3 months of testing. A total of 25 healthy control participants without a history of neurological or psychiatric illness and normal visual acuity were recruited. Further details can be found in Table 1. All participants gave their informed consent prior to completing the study. All ethical procedures complied with the ethical standards of the relevant institutional committees (Melbourne Health Human Research Ethics Committee, 2007.094; Monash University Human Research Ethics Committee, 2017.8068) and the Helsinki Declaration of 1964 and its later amendments.

### 2.2. Study Design

This was a prospective, longitudinal study conducted over a 2-year observational period; baseline, +1 year, +2 years. Average times between visits for baseline to +1 year were 11.35 months (SD = 1.73) and +1 year to +2 years, 11.21 months (SD = 1.72). Total attrition across the study period was 14.29% (*n* = 9).

#### Clinical Tests: Modifying Factors

Tests of depression (Beck Depression Inventory, BDI), premorbid intelligence (National Adult Reading Test, NART), disability (EDSS) and disease duration (months since first symptom) were collected at each study visit (intelligence at baseline only), with tests administered according to standardised instructions (see below for details). For all tests except intelligence, higher values indicated worse performance/severity.

### 2.3. Tests of WM

The following tests were administered according to standardised instructions at each study visit (baseline, +1 year, +2 years). For the ocular motor n-back test, setup and administration were conducted in accordance with the published methodology as outlined in [21].

#### 2.3.1. Episodic Buffer: California Verbal Learning Test (CVLT-II)

The CVLT is a comprehensive test of verbal memory, with the immediate recall section providing a test of the episodic buffer subcomponent of WM [2,19,25].

#### 2.3.2. Phonological Loop: Digit Span Forwards

Digit span forwards requires reciting a number sequence in the same order as verbally presented and provides a test of the phonological loop subcomponent of WM [19,26].

#### 2.3.3. Central Executive: Digit Span Backwards

Digit span backwards requires reciting a number sequence in the reverse order as verbally presented and provides a test of the central executive subcomponent of WM [19,26].

#### 2.3.4. Visual–Spatial Sketchpad: Ocular–Motor n-Back Test

The ocular–motor n-back test provides a test of the visual–spatial sketchpad subcomponent of WM and requires participants to retain visual and spatial information to inform an eye movement response [21,27]. Here, we present data that reflect two WM loads, 0-back, and 1-back, respectively. The tests of interest were response time of a correct response and error. Response time (ms) was calculated as the temporal difference between fixation offset and saccade onset using a velocity criterion of 30° per second, where saccades were initiated >100 ms post cue disappearance and ended within 2·5o of the centre of the correct box. An error was defined as a saccade to an incorrect spatial location or where no attempt to respond was made. The error rate was calculated for each WM load (0-back, 1-back) as a percentage of total trials.

#### 2.3.5. Cognitive Processing Speed: Symbol Digit Modalities Test (SDMT)

The SDMT is a test of attention, processing speed and spatial WM and is considered the gold standard test of cognitive processing speed in pwMS [28].

### 2.4. Data Analysis

IBM SPSS statistics package version 24 was used for all statistical analyses. Baseline differences between groups in EDSS, disease duration (months since first symptom), depression, intelligence and age, were determined using *t*-tests. Pearson’s chi-square analyses were performed to determine group differences in sex and frequency of different WM subcomponent impairment across groups.

All WM test scores were converted to z-scores, where controls represented the normative population, z = $\frac{x-\mu }{\delta }$, and where x = observed value, μ = mean of the normative sample, and σ = the standard deviation of the normative sample. WM impairment was defined as a significant deficit (z-score < −1.5 for the CVLT/SDMT/digit span tests or >1.5 for the OM n-back test) on at least one WM test. Receiver operating characteristics (ROC) statistics were used to determine the diagnostic capacity and cut-off scores for each WM subcomponent test under the null hypothesis of an area under the curve (AUC) of 0.5. Optimal cut-offs for each WM test were selected by the Youden J index: sensitivity + specificity − 1. A Youden J index above 0.5 meets the empirical benchmark for diagnostic ability [29]. Positive and negative predictive values were calculated for each WM test (PPV, NPV), as well as accuracy according to the formula: $\frac{\mathrm{true}\;\mathrm{positives}\;+\;\mathrm{true}\;\mathrm{negatives}}{\mathrm{total}\;\mathrm{sample}}$. Logistic regressions were used to determine probability of WM impairment detection using different permutations of WM tests.

Frequency of single subcomponent WM test failure and multi-subcomponent test failure was calculated using derived optimal cut-off scores. A single subcomponent WM impairment was defined as an individual who performed below cut-off on a single WM test and which corresponded to a z-score < −1.5 for the CVLT/SDMT/digit span tests or >1.5 for the OM n-back test. A multi-subcomponent WM impairment was defined as an individual who performed below cut-offs on two or more WM tests and had a z-score < −1.5 for the CVLT/SDMT/digit span tests or >1.5 for the OM n-back test.

Change in WM performance over time for each WM subcomponent test was determined using linear regression adjusted models. For all models, factors included time (baseline, +1 year and +2 years) and group (CIS, CDMS). For the OM n-back test, an additional factor of WM load (0-back, 1 back) was included. Fixed effects and interactions were generated for each model. A subject-specific random effect was included in all models to account for between-subject heterogeneity. Compound symmetry was set as the repeated covariance type following visualisation of residual plots. Covariates included were age, intelligence (NART), depressive symptomology (BDI), disease duration (months since first symptom(s)) and sex (male, female). Where theoretically relevant effects/interaction were deemed relevant covariates, these were included as fixed effects. All models were based on 344 data points for the OM n-back test and 174 data points for the CVLT, SDMT and digit span tests.

Reliable change indices (RCIs) were calculated to determine the proportion of early RRMS whose performance had significantly deteriorated (*p* < 0.05) between baseline and +2 years. Calculation of RCIs was conducted in accordance with the Jacobson–Truax index procedure [30]: $\frac{x_{1}-x_{2}}{S_{diff}}$, *x*_1_ = baseline WM performance, *x*_2_ = +2 years WM performance, *S_diff_* = $\sqrt{2{\left(S_{E}\right)}^{2}}$ and *S_E_* = standard error of measurement. For the CVLT/SDMT/digit spans, an RCI score of <−1.5 indicated significant (*p* < 0.05) deterioration in performance from baseline; a z-score of >1.5 was used for OM n-back.

## 3. Results

### 3.1. Descriptive Information for Early RRMS and Healthy Controls

Early RRMS patients (CIS and CDMS) were not significantly different from healthy controls in terms of age, depressive symptomology (BDI) or premorbid intelligence (NART). Overall, CIS patients were significantly younger than CDMS patients (F(1, 63) = 7.54, *p* = 0.008) and had a significantly shorter disease duration (F(1, 44.96) = 28.67, *p* < 0.000); EDSS was comparable between CIS and CDMS groups. All participants exhibited visual acuity within the normal range, either corrected or uncorrected (6/4–6/6) (Table 1).

### 3.2. Diagnostic Accuracy of WM Tests for Identifying WM Impairment

Some 61.9% of early RRMS patients had a WM impairment. The CVLT, DS forward, DS backward and n-back (error) demonstrated an AUC significantly greater than 0.5, indicating diagnostic accuracy significantly greater than chance for identifying WM impaired patients. However, only n-back (error) had a Youden J index above 0.5, meeting the empirical benchmark for diagnostic purposes. The n-back (error) and DS forward demonstrated the highest accuracy of around 70% for correctly identifying patients with and without WM impairment. See Table 2.

### 3.3. Frequency of WM Test Failure

**Figure 1:**
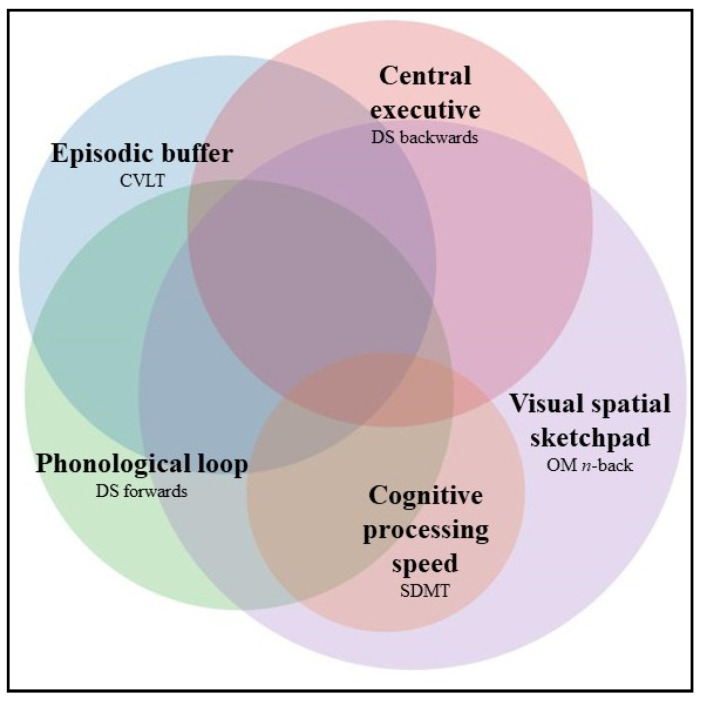
Proportion of early RRMS who failed each WM subcomponent test at baseline. Size of circles indicates the proportion of early RRMS patients who performed below cut-off scores in each WM test. The degree of overlap between tests (darker areas) represents the proportion of RRMS patients who failed multiple WM tests. Regions: OM n-back (70.5%), SDMT (18.2%), DS forward (43.2%), DS backwards (38.6%) and CVLT (40.9%). Overlap regions: OM n-back*SDMT (50%), OM n-back*DS forwards (29.5%), OM n-back*DS backwards (27.3%), OM n-back*CVLT (18.2%), DS forwards*SDMT (9.1%), DS backwards*SDMT (2.3%), DS forwards*CVLT (22.7%), DS backwards*CVLT (18.2%), OM n-back*SDMT*DS forward (9.1%), OM n-back*SDMT*DS backwards (4.5%) and OM n-back*CVLT*DS forwards (18.2%). OM n-back*CVLT*DS backwards (15.9%). Percentages expressed as proportion of total patients impaired: *n* = 44.

Based on optimal derived cut-off scores, 69.8% of early RRMS patients failed at least one WM test, with 70.5% failing the OM n-back (visual–spatial sketchpad), 43.2% failing DS forwards (phonological loop), 40.9% failing the CVLT (episodic buffer), 38% failing DS backwards (central executive) and 18.2% failing the SDMT (cognitive processing speed). Of these, 38.6% failed a single WM test, and 61.4% failed multiple WM tests. Figure 1 depicts the frequency of each WM subcomponent test failed and the degree of overlap between failure on different WM test.

### 3.4. Frequency of Single and Multi-Subcomponent WM Impairment

Of the 38.6% of patients who failed a single WM test, 76.4% were classified as impaired on the failed test (i.e., single subcomponent impairment). The most common single WM subcomponent impairment was associated with the visual–spatial sketchpad (OM n-back), followed by the episodic buffer (CVLT), the phonological loop (DS forwards) and the central executive (DS backwards) Figure 1.

**Figure 2:**
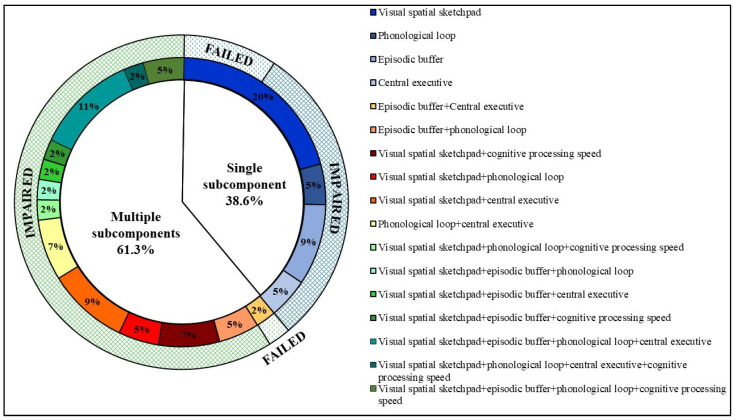
Frequency of WM subcomponent test failure and proportion of patients who were impaired on a single or multiple WM subcomponents. IMPAIRED: refers to a patient with early RRMS who performed below optimal cut-off scores on either a single WM test or multiple WM tests that corresponded to a z-score < −1.5 for the CVLT/SDMT/DS tests or >1.5 for the OM n-back test. FAILED: refers to a patient with early RRMS who performed below cut-off on either a single WM test or multiple WM tests that corresponded to a z-score > −1.5 for the CVLT/SDMT/DS tests or <1.5 for the OM n-back test.

Of the 61.4% of patients who failed multiple WM tests, 96.3% were classified as impaired on each test failed (i.e., multi-subcomponent impairment). The three most common multi-subcomponent impairments were (1) visual–spatial sketchpad + episodic buffer + phonological loop + central executive, (2) visual–spatial sketchpad + central executive and (3) visual–spatial sketchpad + cognitive processing speed. Full details can be found in Figure 2.

### 3.5. Diagnostic Accuracy for Combination of WM Subcomponent Tests for Identifying WM Impairment

To improve the diagnostic sensitivity and specificity, WM tests were examined in different combinations to determine the most parsimonious battery of WM tests whilst balancing the AUC, sensitivity and specificity.

All WM test combinations demonstrated an AUC significantly greater than 0.5, indicating diagnostic accuracy significantly greater than chance for identifying WM-impaired patients. The inclusion of all four WM tests produced the highest AUC (0.81). However, the highest AUC for three WM tests (CVLT + DS forwards + OM n-back, AUC = 0.80) and two WM tests (DS forwards + OM n-back, AUC = 0.78) indicated similar diagnostic accuracy to when all WM tests were included. See Table 3.

### 3.6. Change in WM Subcomponent Performance over Two Years (Baseline, +1 Year, +2 Years)

All adjusted means and standard deviations for each WM subcomponent test and clinical covariates at each study visit can be found in Table 4.

#### 3.6.1. Visual–Spatial Sketchpad: OM n-Back Test

##### Error

A significant effect of time was found (F(2, 144.90) = 3.41, *p* = 0.036), with the error rate significantly reducing on average by 6.70% (SE = 3.4) across the three study visits: 5.10% (SE = 2.89) between baseline and +1 year, 0.17% (SE = 2.62) between +1 year and +2 years. A measure of time by group interaction (F(2, 143.18) = 3.45, *p* = 0.034) revealed that the significant reduction in error was only present for the CDMS group (df (2, 122.38) = 11.79, *p* = 0.000021: baseline—+1 year mean difference = 13.35 (SE = 0.42), df = 164.63, *p* = 0.000015, 95% CI: 6.52, 20.19; baseline—+2 years mean difference = 10.65 (SE = 0.44), df = 135.04, *p* = 0.0001, 95% CI: 3.83–17.47). Two early RRMS (1 CIS, 1 CDMS) showed a significant worsening in error rate.

##### Response Time

In addition to the standard covariates, the error *RRMS subgroup* time was included to account for practice effects and the possibility of a speed/accuracy trade-off; the significant reduction in error rate over time raises the possibility that the increase in response time may be a consequence of patients sacrificing speed (prolonging response time) to increase accuracy (reducing error rate). A significant effect of time was evident (F(2, 141.32) = 22.73, *p* = 2.76 × 10^−9^), with response time found to increase on average 188.89 ms (SE = 29.10) across the three study visits; 122.68 ms (SE = 24.59) between baseline and +1 year, 66.21 ms (SE = 23.37) between +1 year and +2 years. A significant RRMS subgroup by time interaction was also evident (F(2, 138.18) = 5.59, *p* = 0.005), with both CIS (average mean difference = 291.77 (SE = 39.89), df(2, 149.49) = 14.84, *p* = 0.000001) and CDMS groups (average mean difference = 86.01 (SE =5.26), df(2, 128.03) = 8.14, *p* = 0.00047) demonstrating a significant increase in response time. Only the CIS group demonstrated a significant increase between each consecutive time point (baseline—+1 year mean difference = −161.21 (SE = 42.87), df = 169.72, *p* = 0.001, 95% CI: −264.87, −57.56; +1 year—+2 years mean difference = −130.56 (SE = 42.54), df = 106.65, *p* = 0.008, 95% CI: −234.07, -27.04), while the CDMS group only demonstrated a significant increase between baseline and +1 year (mean difference = −84.16 (SE = 24.59), df = 156.22, *p* = 0.002, 95% CI: −143.67–−24.64). In total, 15 early RRMS (23.81%: 5 CIS, 10 CDMS) showed significant slowing of response time.

#### 3.6.2. Episodic: CVLT

A significant effect of the RRMS subgroup was evident (F(1, 91.11) = 7.48, *p* = 0.007), with the CDMS group performing poorer than the CIS group (mean difference = 43.37 (SE = 14.46), df (1, 95.06) = 9.02, *p* = 0.003, 95% CI: 14.50, 72.05). A significant RRMS subgroup by time interaction was found (F(2, 57.79) = 7.43, *p* = 0.001), with the CDMS subgroup alone demonstrating an average of 6.67 (2.64) point improvement over time (df (2, 71.86) = 3.62, *p* = 0.032); significant improvement occurred between baseline and +2 years only (df = 63.94, *p* = 0.037, 95% CI: 13.21, 0.31). Only one early RRMS group (CDMS) showed significant reduction in performance.

#### 3.6.3. Phonological Loop: DS (Forwards)

No significant effects or interactions were evident. In total, three early RRMS (CDMS) showed a significant reduction in performance.

#### 3.6.4. Central Executive: DS (Backwards)

No significant effects or interactions were evident. In total, two early RRMS (CDMS) showed a significant reduction in performance.

#### 3.6.5. Cognitive Processing Speed: SDMT

A significant effect of the RRMS subgroup was found F(1, 88.10) = 6.01, *p* = 0.016). However, no significant difference between RRMS subgroups was evident. No other significant effects or interactions were evident. No individual RRMS was found to have a significant reduction in performance across the study period.

## 4. Discussion

The aim of this study was to determine (1) the WM phenotype(s) most frequently affected in early RRMS and whether phenotypes most commonly implicate single or multiple subcomponents, (2) how different WM subcomponents progress over time, accounting for individual and disease modifying factors, and (3) which test(s) provide the best diagnostic measure of WM impairment and measures progression.

### 4.1. Frequency of WM Subcomponent Test Failure and WM Phenotypes in Early RRMS

WM impairment was found in over 60% of this early RRMS cohort. The most commonly implicated WM subcomponent was the visual–spatial sketchpad (70.5% failure rate) followed by the phonological loop (43.2% failure rate), the episodic buffer (40.9% failure rate) and the central executive (38% failure rate). In contrast, cognitive processing speed as measured by the SDMT was only implicated in 18% of patients. This is in line with previous research in early MS that has shown that visual–spatial and phonological WM are most frequently impaired [21,22].

Interestingly, the majority of patients with a WM impairment had concomitant impairments in multiple WM subcomponents (59.1%) as opposed to just a single WM subcomponent (29.5%). While there was variability in the type of WM phenotype, the visual–spatial sketchpad was the most commonly affected in both single and multi-subcomponent impairments. Furthermore, the frequency of patients reaching the impairment threshold as opposed to just test failure was much higher when multiple subcomponents were implicated as opposed to just a single subcomponent. This is similar to general cognitive impairment, with increasing disease severity related to increasing number of cognitive domains impaired [1,31,32,33].

### 4.2. Diagnostic Accuracy of WM Subcomponent Tests: Recommendation for Test Selection in Early RRMS

All WM tests demonstrated diagnostic accuracy significantly greater than chance in identifying patients with a WM impairment. In contrast, the SDMT did not achieve diagnostic accuracy greater than chance. Although this may be unsurprising given that the SDMT is a measure of cognitive processing speed in MS, this does highlight the limitation of the SDMT as a general screening tool for cognitive impairment in early MS.

The OM n-back (visual–spatial sketchpad) and the DS forwards (phonological loop) both demonstrated the highest levels of sensitivity, specificity and accuracy for diagnosing WM impairment in patients independently. The auditory system, particularly central auditory processing that subserves the phonological loop [34], and the visual system that subserves the visual–spatial sketchpad is known to be affected in MS [35,36], and often present in early disease [37]. Furthermore, impairments within these systems have been shown to be related to reduced performance on neuropsychological tests that rely upon them [38,39,40,41]. However, only the OM n-back achieved a Youden index greater than 50% and thus met the empirical benchmark for diagnostic purposes.

Importantly, all WM tests, including the OM n-back and DS forward, had relatively low individual sensitivity for diagnosing WM impairment. This is likely a consequence of the definition of WM impairment used (impairment on at least one test of WM) and the specificity of these tests to detect change within a single WM subcomponent. This highlights the precariousness of assessing WM impairment in early RRMS patients using only a single test, with the likelihood of detecting impairment low unless the correct test is used.

While the inclusion of all four WM tests provided the highest diagnostic probability of detecting patients with WM impairment and improved sensitivity, this was negligible to when only the OM n-back and DS forward were combined. This suggests that these two tests in combination may be sufficient to use in early RRMS to detect the majority of patients with WM impairment.

Finally, where a single test is required, assessment of the visual–spatial sketchpad (OM n-back) or the phonological loop (DS forwards) alone provided approximately 70% accuracy in detecting early RRMS patients with or without WM impairment. However, as mentioned, only the OM n-back had a Youden J index above 50%, meeting the empirical threshold for diagnostic purposes. Furthermore, approximately half of the patients who failed the OM n-back did not reach impairment levels, suggesting that this test may have the capacity to detect patients at risk of future impairment.

### 4.3. Progression in WM Subcomponent over Two Years

A significant deterioration in performance was only evident in response times on the visual–spatial sketchpad test (OM n-back), with approximately 24% of early RRMS exhibiting a significant increase in response time across the study period, independent of an improvement in error rate. All other WM subcomponent tests were stable or significantly improved (episodic buffer—CVLT); less than 7% of early RRMS worsened over the two-year study period. This disparity in findings suggests different rates of progression for the different subcomponents of WM, and/or differences in the sensitivity of the tests used and their capacity to detect meaningful change within a two-year period.

The significant increase in response time for the OM n-back test reflects a reduction in processing speed associated with the transmission of visual–spatial information to, and processing within, the visual–spatial sketchpad; it does not reflect an inability to maintain and utilise visual–spatial information. Reduced processing speed within the visual–spatial WM network and the broader ocular motor network that is proposed to mediate its proficiency [42,43] is frequently found in early RRMS and worsens commensurate to disease duration [21,32,44,45,46,47,48,49]. Furthermore, a study by Huijbregts et al. [50] found that RRMS patients are more vulnerable on tests that place demands concurrently on processing speed and visual–spatial working memory, such as the OM n-back test.

Interestingly, in our study, we did not see a commensurate worsening in performance on the SDMT, the proposed gold standard test of processing speed in MS [28,51] that similarly places demands on visual–spatial WM and processing speed [52,53]. This is likely due to the sensitivity of the SDMT to detect WM impairment and progression within a two-year time frame in early RRMS. Previous studies have similarly found that the ability of the SDMT to detect cognitive deterioration within 2–3 years is poor [54,55], with a large study of 531 MS patients reporting that SDMT performance is estimated to decrease by an imperceptible 0.22 points per year [56]. The poor sensitivity of the SDMT and the other WM tests that demonstrated no change or improvement (DS forwards, DS backwards, CVLT) in the study period is likely a consequence of the psychometric properties of the tests and the influence of practice effects. This questions the utility of these tests to measure progression in early RRMS within a two-year period.

Practice effects are a known issue with repeated administration of neuropsychological assessments and manifest as an improvement in performance or the absence of change [57,58,59]. Indeed, in early RRMS, evidence suggests that progression only becomes evident once impairment is greater than the masking effect of practice [50,60]. Broadly, practice effects are a consequence of the unavoidable implication of long-term memory that occurs naturally upon engagement in a test or activity. This engagement may result in long-term consolidation of test-specific items due to rehearsal, general test familiarity and/or the development and retention of memory devices such as grouping individual pieces of information into larger units (chunking) [61]; this device is commonly used on the CVLT (classifying related words into groups: animals, transport, vegetable, furniture) [62], and digit span tests (grouping of individual numbers into large numbers) [63]. While the OM n-back test was not free from practice effects (i.e., improvement in error rate was seen), this did not impact the detection of response time slowing. OM assessments are performed using high-powered cameras (sample rate of 500 Hz) that necessarily afford the precision to detect and delineate subtle yet significant incremental increases in response time. Indeed, previous work published by our group has demonstrated that OM assessments consistently detect impairments in early RRMS not evident on common neuropsychological tests of cognitive processing speed used in MS [21,45,46,47,64,65]. Furthermore, unlike the other WM tests used in this study, OM tests employ a multiple repeated trial designs that is thought to underlie the high test–retest reliability reported in other studies [66,67,68]. In combination, these psychometric properties likely mitigate the impact of practice effects on OM n-back response times, allowing the detection of significant progression of impairment.

### 4.4. Limitations

Firstly, it needs to be acknowledged that other WM subcomponents, namely those ubiquitous WM subcomponents (central executive and episodic buffer) are likely to interact with other WM subcomponents. Thus, impairments attributed to an individual subcomponent may in some individuals reflect impairments in these other subcomponents. Future studies using magnetic resonance imaging to determine brain regions activated during test performance would allow further demarcation of WM subcomponent impairments in early RRMS. Secondly, the absence of a 2-year follow-up in our control population prohibits the clarification of normal change in WM subcomponents over the study period. Indeed, response times are known to increase as a factor of time/age. However, age-related response time slowing is known to be subtle, with one large study reporting that response time slowed on average by only 1.6 ms per year [69]. Thus, the 188.89 ms slowing in visual–spatial response time found in this study is reasonably interpreted as abnormal and the consequence of MS disease. Inclusion of other measures of processing speed, such as the newly validated Letter Digit Substitution Test in MS, may help to elucidate these changes [70]. Finally, the inclusion of VEP and AEP investigations would have allowed determination of whether these changes are related to and/or impact the type of WM subcomponents impacted. This is an important area of future research.

## 5. Conclusions

WM impairments are a frequent symptom of early RRMS primarily associated with the visual–spatial sketchpad and the phonological loop. When WM impairment is present, it is more likely to present as a multi-subcomponent phenotype and require a multi-test approach to achieve the best diagnostic accuracy. Furthermore, findings also demonstrated the limitations of the SDMT to detect WM impairment in early RRMS, an important consideration given the proclivity for using the SDMT as a single screening tool for cognitive impairment in MS. Finally, the OM n-back demonstrated the highest diagnostic accuracy and sensitivity to both WM impairment and progression. Given the superior technological precision associated with OM assessment, these results highlight the utility of using more sophisticated assessment procedures when assessing MS patients with more mild disease.

## Figures and Tables

**Table 1:** Demographic and clinical information for early RRMS and healthy controls.

	Healthy Controls (*n* = 25)		Early RRMS					
			CIS (*n* = 14)		CDMS (*n* = 49)		Total Early RRMS (*n* = 63)	
	M (SD)	Range	M (SD)	Range	M (SD)	Range	M (SD)	Range
Age (years)	38.63 (11.07)	21–65	33.00 * (8.38)	20–46	42.18 * (11.64)	19–66	40.14 (11.60)	19–66
Gender F (M)	21 (3)	-	12 (2)	-	45 (4)	-	57 (6)	-
NART	117.00 (4.23)	110–124	115.29 (5.14)	105–123	115.92 (4.56)	106–125	115.78 (4.65)	105–125
BDI	3.95 (3.29)	0–14	6.85 (5.36)	1–18	6.67 (8.19)	0–38	6.71 (7.61)	0–38
EDSS			0.00 (0.00)	0–2	0.00 (1.00)	0–3.5	0.00 (1.00)	0–3.5
Disease duration (months)	-	-	12.85 ** (11.72)	2–37	104.54 ** (110.75)	4–513	83.43 (104.54)	2–513

NART: National Adult Reading Test; BDI: Beck’s Depression Inventory; EDSS: Expanded Disability Severity Scale for EDSS; results are expressed as median (interquartile range); Disease duration: months since first symptom(s). Significantly different between MS subgroups at * *p* < 0.05 or ** *p* < 0.001 significance.

**Table 2:** Diagnostic accuracy of WM tests.

WM Test	AUC (95% CI)	Cut-Off Scores	Youden J	Sensitivity (Specificity) (%)	PPV (%)	NPV (%)	Accuracy (%)
CVLT Episodic buffer	0.66 (0.53–0.79) *	34.49	39.24	43.6 (95.7)	61.1	50	62.9
SDMT Cognitive processing speed	0.53 (0.39–0.68)	51.51	20.51	20.5 (100)	100	56.6	50.8
Digit span-Forwards Phonological loop	0.71 (0.59–0.83) *	9.50	48.72	48.7 (100)	100	46.5	67.7
Digit span-Backwards Central executive	0.69 (0.56–0.82) *	5.50	29.77	38.5 (91.3)	88.2	53.3	58
OM n-back Visual–spatial sketchpad							
Response time (ms)	0.52 (0.38–0.66)	674.46	25.00	33.3 (91.2)	86.6	54.2	55.5
Error rate (%)	0.73 (0.60–0.85) *	26.10	51.68 +	53.8 (95.8)	95.5	43.9	70.1

* *p* < 0.05; + Youden J > 50 indicates diagnostic sensitivity [29]; AUC: Area Under the Curve; PPV: Positive Predictive Value; NPV: Negative Predictive Value.

**Table 3:** Diagnostic accuracy of different WM test combinations.

	AUC	Cut-Off Probability	Youden J	Sensitivity (%)	Specificity (%)
Two WM tests					
DS backwards + DS forwards	0.754 *	0.7	52.84	61	91
CVLT + DS backwards	0.755 *	0.71	47.71	56	91
CVLT + DS forwards	0.760 *	0.72	57.19	61	95
DS backwards + OM n-back	0.762 *	0.76	53.62	66	87
CVLT + OM n-back	0.775 **	0.49	48.72	48	100
DS forwards + OM n-back	0.784 **	0.72	54.63	59	85
Three WM tests					
CVLT + DS forwards + DS backwards	0.786 **	0.71	62.32	66	95
DS backwards + DS forwards + OM n-back	0.789 **	0.69	57.19	61	95
CVLT + DS backwards + OM n-back	0.798 **	0.65	57.97	66	91
CVLT + DS forwards + OM n-back	0.802 **	0.77	58.97	59	100
Four WM tests					
CVLT + DS forwards + DS backwards + OM n-back	0.810 **	0.75	58.97	59	100

* *p* < 0.01, ** *p* < 0.001.

**Table 4:** Adjusted averages for WM subcomponent tests and clinical covariates across the study visits.

	Early RRMS								
	CIS M (SE)			CDMS M (SE)			Total Early RRMS M (SE)		
WM Tests	Baseline *n* = 14	+1 Year *n* = 14	+2 Year *n* = 12	Baseline *n* = 49	+1 Year *n* = 45	+2 Year *n* = 42	Baseline *n* = 63	+1 Year *n* = 59	+2 Year *n* = 54
CVLT Episodic buffer	89.20 (15.75)	79.72 (14.28)	80.63 (12.25)	40.12 (1.88)	42.38 (2.46)	46.97 (1.91)	64.66 (7.93)	61.05 (7.24)	63.80 (6.21)
SDMT Cognitive processing speed	61.64 (14.63)	67.25 (12.84)	71.93 (12.09)	64.04 (1.71)	66.91 (1.90)	67.59 (2.81)	62.84 (7.37)	67.08 (6.49)	69.76 (6.21)
Digit span-Forwards Phonological loop	15.65 (2.84)	15.60 (2.50)	16.90 (2.10)	11.19 (0.34)	11.17 (0.41)	11.36 (0.36)	13.42 (1.43)	13.39 (1.27)	14.13 (1.07)
DS-Backwards Central executive	10.21 (2.67)	9.57 (2.33)	10.44 (1.97)	6.97 (0.34)	7.59 (0.36)	7.67 (0.33)	8.59 (1.34)	8.58 (1.18)	9.05 (1.00)
OM n-back Visual–spatial sketchpad									
Response time (ms)	539.80 (111.44)	671.56 (119.25)	703.00 (147.70)	600.40 (152.97)	667.31 (120.19)	672.46 (117.91)	586.94 (146.17)	668.26 (118.63)	677.55 (122.52)
Error rate (%)	17.15 (10.70)	15.04 (11.06)	16.48 (25.12)	25.26 (20.98)	19.06 (20.53)	18.96 (14.42)	23.46 (20.18)	19.61 (20.53)	18.55 (16.65)
Clinical covariates									
BDI	4.8 (1.23)	3.00 (1.22)	4.4 (1.08)	6.29 (1.79)	6.19 (1.32)	4.48 (0.85)	6.71 (7.61)	6.95 (7.73)	5.88 (6.64)
EDSS Mdn	0 (0)	0.3 (0.20)	0.3 (0.20)	0.57 (0.27)	0.67 (0.28)	0.67 (0.28)	0.29 (0.14)	0.48 (0.24)	0.48 (0.24)
Disease duration (m)	15.4 (6.64)	27.00 (6.52)	39.80 (5.88)	125.90 (30.99)	137.43 (31.01)	149.28 (30.99)	83.43 (104.54)	95.70 (106.17)	114.98 (107.41)
Age	34.8 (3.73)	35.80 (3.73)	37.00 (3.73)	42.48 (2.30)	43.43 (2.70)	44.38 (2.70)	40.14 (11.60)	41.64 (11.34)	42.94 (11.34)
Sex F(M)	12 (2)	12 (2)	10 (2)	45 (4)	43 (4)	38 (4)	57 (6)	53 (6)	48 (6)
NART+	115.29 (5.14)	-	-	115.92 (4.56)	-	-	115.78 (4.65)	-	-

WM: working memory; CVLT: California Verbal Learning Test II; SDMT: Symbol Digit Modalities Test; OM: Ocular Motor; BDI: Beck Depression Inventory; EDSS: Expanded Disability Severity Scale; NART: National Adult Reading Test (Pre-morbid intelligence). +NART was administered once at baseline, in line with test instructions, with the same score used as a covariate at each study visit.

## Data Availability

Data has not been made available for this study.
